# Effectiveness of a Virtual Exercise Program During COVID-19 Confinement on Blood Pressure Control in Healthy Pregnant Women

**DOI:** 10.3389/fphys.2021.645136

**Published:** 2021-03-10

**Authors:** Cristina Silva-Jose, Miguel Sánchez-Polán, Ángeles Diaz-Blanco, Javier Coterón, Ruben Barakat, Ignacio Refoyo

**Affiliations:** ^1^Actividad Físico-Deportiva en Poblaciones Específicas (AFIPE) Research Group, Facultad de Ciencias de la Actividad Física y el Deporte, Universidad Politécnica de Madrid, Madrid, Spain; ^2^Servicio de Obstetricia y Ginecología, Hospital Universitario Severo Ochoa, Leganés, Madrid, Spain

**Keywords:** exercise, pregnancy, maternal, gestational hypertension, pandemic

## Abstract

**Background:**

The situation caused by COVID-19 has led to movement restrictions for the majority of the population due to the confinement established by the health authorities. This new situation has changed people’s habits and significantly affected the pregnant population. Decreased exercise and increased psychophysical stress are associated with excessive weight gain, diabetes, and gestational cardiovascular complications that affect the mother, fetus, and newborn. Recent research shows that the dynamics of maternal blood pressure is one of the most important control factors during pregnancy. Thus, prevention of these type of pathologies through interventions without maternal-fetal risks is important.

**Objectives:**

To examine the influence of a virtual exercise program on maternal blood pressure during pregnancy.

**Materials and Methods:**

A randomized clinical trial design was used (NCT04563065). Data from 72 pregnant women without obstetric contraindications under confinement conditions in the Madrid area were collected. Women were randomly assigned to the intervention (IG) or control group (CG). They previously signed informed consent forms. A moderate exercise program was performed as an intervention from 8–10 to 38–39 weeks of pregnancy. Systolic (SBP) and diastolic (DBP) maternal blood pressure were measured during the first, second and third trimesters of pregnancy, as well as before and immediately after delivery in both study groups.

**Results:**

No differences in systolic and diastolic blood pressure during the first, second and third trimesters were found between groups. Significant differences in SBP were found immediately before delivery (IG = 119.83 ± 10.16 vs. CG = 125.6 ± 10.91; *p* = 0.047) and immediately after delivery (IG = 115.00 ± 11.18 vs. CG = 122.24 ± 15.71; *p* = 0.045).

**Conclusions:**

Results show lower SBP values for the IG during delivery than CG. A virtual exercise program throughout pregnancy during COVID-19 confinement can help to control systolic blood pressure before and immediately after delivery in healthy pregnant women.

## Introduction

Given the quantity and quality of modifications in a woman’s body that are involved in pregnancy, there is no physiological process like pregnancy or childbirth in the life of the human (Barakat et al., 2015). Pregnancy is distinguished by a multitude of physiological, mental and emotional adjustments. Every organ system in the expectant mother is intimately involved in this complex process, to create an optimal environment for fetal development (Artal et al., 1991). From the circulatory/hemodynamic point of view, the changes begin during the fifth week of gestation and last until approximately 1 year after delivery (Clapp and Capeless, 1997). Cardiac output increases by approximately 40% (due to an increased stroke volume and heart rate [HR]). There is a 13% increase in body mass supplied by the maternal blood (Geva et al., 1997). Important changes in the blood volume, systemic vascular resistance and vascular tone exist during pregnancy (Gilson et al., 1997; Carbillon et al., 2000; Bamfo et al., 2007; Zentner et al., 2012).

An increasing number of women in Western countries develop cardiovascular disease during pregnancy. The estimation is a rise of risk about 0.2–4% (Perales et al., 2016). Hypertension is one of the most common gestational complications with a prevalence of 10% depending on factors such as country and population studied, and the criteria used to establish the diagnosis (Acog Committee on Practice Bulletins–Obstetrics, 2002; Obstetrics and Gynecology, 2013, 2019). The consequences of cardiovascular disorders affect the mother, fetus, and newborn. Indeed, hypertensive complications during pregnancy remain a leading cause of maternal and neonatal morbidity and mortality (Amro and Sibai, 2020) and may remain 5–15 years following delivery (Bellamy et al., 2007). Many studies have shown that a sedentary lifestyle and decreased movement are determinants of multiple deleterious complications, especially cardiovascular complications (Rand et al., 2020; Yong et al., 2020).

Due to the serious health problem presented by the current global pandemic, the movement restrictions and confinement caused by the COVID-19 pandemic have significantly affected the lifestyles of the pregnant population and can become a risk factor for different alterations and even pathologies (Ayaz et al., 2020; Juan et al., 2020; Moyer et al., 2020; Zaigham and Andersson, 2020). In the current sanitary situation, the new confinement and environment of decreased physical mobility can increase the risks of cardiovascular disease, such as gestational hypertension, with several associated complications (Alomari et al., 2020; Justman et al., 2020; Magee et al., 2020).

Furthermore, the social isolation experienced by pregnant women during confinement has kept them away from their family members and other networks, and consequently this has led to an increase in prenatal depressive symptoms and anxiety (Ammar et al., 2020a,b; Chivers et al., 2020; Durankuş and Aksu, 2020); this implies an essential need for strategies to support both physical and mental health of pregnant women.

The prevention of these pathologies should be a basic column in the sanitary planning of health institutions, and it is necessary to use innocuous interventions that achieve this prevention without adverse effects for the mother, fetus, and newborn. The use of exercise as one of those preventive columns in general populations is sufficiently supported by scientific evidence; however, the efficacy of exercise in this prevention strategy during pregnancy is still poorly investigated (Davenport et al., 2018). In that sense, several scientific studies confirm the relationship between exercise during pregnancy and improved pregnancy outcomes. Therefore, international guidelines for exercise during pregnancy recommend an active pregnancy for pregnant women without obstetric complications (Mottola et al., 2018; Barakat et al., 2019; Obstetrics and Gynecology, 2020). In this sense, with the continuity of the pandemic and lack of face-to-face activities, one strategy that could be is delivery of physical activity interventions through virtual modalities.

The main objective of this study was to examine the influence of a virtual supervised exercise program throughout pregnancy on maternal systolic blood pressure (SBP) and diastolic blood pressure (DBP). We hypothesize that a supervised, moderate, and regular exercise program throughout pregnancy may be a helpful factor in controlling maternal blood pressure.

## Materials and Methods

### Study Design

This study was developed by the collaboration between the Obstetrics and Gynecology Department of the Hospital Universitario Severo Ochoa (Madrid) and Universidad Politécnica de Madrid. A randomized clinical trial (NCT04563065) was approved by the Ethical Commission of Research of Universidad Politécnica de Madrid. Women were randomly assigned to the intervention group (IG) or control group (CG). The Selene program at the Hospital Universitario Severo Ochoa in Leganés (Madrid) was used to collect the personal, labor, and medical data of the participants.

### Participants and Randomization

**FIGURE 1:**
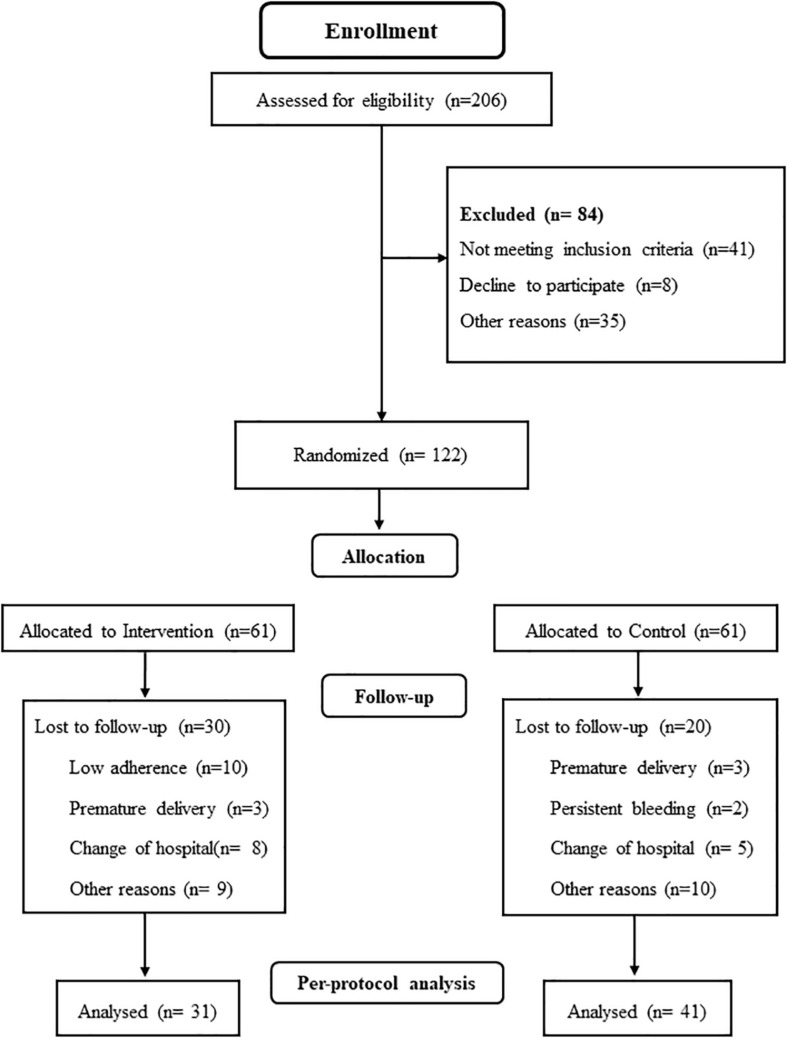
Study population flow chart.

A total of 206 Spanish-speaking pregnant women from hospital obstetric consults (Figure 1) were assessed for eligibility. Women aged between 18 and 45 years with singleton and uncomplicated pregnancies, with no history or risk of preterm delivery and not participating in any other trial or exercise program were invited to participate. The following conditions were excluded of the study: not planning to give birth in the same obstetric hospital, not being under medical follow-up throughout pregnancy having any serious contraindicated conditions for practicing safe exercise (Mottola et al., 2018; Barakat et al., 2019; Obstetrics and Gynecology, 2020).

For the randomization of participants, a process of allocation concealment by random number blocks was used. Assessment staff was blinded to the assignments. Randomization process (sequence generation, allocation concealment, and implementation) was conducted by three different individuals.

#### Intervention

Women assigned to the intervention group (IG) adhered to a virtual supervised exercise program between 8–10 and 38–39 weeks of pregnancy. An average of 80–85 training sessions were originally planned for each participant, and a minimum of 80% adherence to the exercise program was required to be included in the analysis of the results.

The virtual supervised exercise program involved 3 weekly sessions of 55–60 min of varied activities following a model established by our research group. Regarding the moderate intensity of the workload and due to the non-face-to-face nature of the program, pregnant women were previously informed for self-control through two mechanisms: Maternal Heart Rate (MHR) and perception of effort. Therefore, women used a heart rate monitor (Accurex Plus, Finland) during the training sessions (MHR was consistently 55–65% of heart rate reserve using the Karvonen formula) and a range of 12–14 of Borg Rate of Perceived Exertion Scale (Somewhat Hard) (Barakat, 2020).

From the methodological point of view, the session was divided into seven parts (Barakat, 2020):

1. Warm-up with general exercises of 5–7 min: Range of motion varied, but impact activities were not included (avoidance of jumps and falls).
2. Aerobic exercises of 8–10 min: Exercises were performed to increase the intensity up to that of moderate activities.
3. Muscle strengthening and general toning exercises of the whole body of 10–12 min: Exercises for the lower extremities (calf, quadriceps, hamstrings, adductors, abductors) and torso (abdominal, pectoral, shoulders, paravertebral musculature) were included. The muscle groups to train were distributed into the 3 weekly sessions. During each session, one or two sets of 10–12 repetitions were performed from each muscle group using barbells (2–3 kg/exercise) or low-to-medium resistance (elastic) bands (Therabands). Exercises for the most weakened muscle groups during pregnancy were also included, as the aim was to avoid muscular decompensation.
4. Coordination and balance exercises of 5–8 min: Simple eye-hand and eye-foot coordination tasks were performed with sports equipment, as well as body axis balance exercises.
5. Strengthening the pelvic floor muscles of 8–10 min: Kegel exercises were performed.
6. Cool down session of 5–7 min: The aim was to gradually lower the intensity of work with flexibility, stretching, and relaxation exercises.
7. Final discussion of 7–8 min: The aim of this session was for the pregnant women to clearly and openly express their sensations and perceptions experienced during the training session. This part was conducted only during the group virtual session.

Exercises in the supine position were not performed for more than 2 min and impact activities were not included in the sessions. Adequate hydration before and after exercise was recommended and high humidity and temperature environment for exercise was avoided in order to prevent maternal hyperthermia.

The exercise program was provided by two modalities:

1. Individual work (2 weekly sessions): These were recorded sessions, with complete visual information and indications regarding the exercises to be performed. These sessions were designed so that pregnant women could follow very easily and intuitively, and the participants had simple and agile access for downloading.
2. Group work (1 weekly session): Classes were supervised online through the Zoom Video platform.

#### Usual Care (Control) Group

Women assigned to the CG received general advice from their health care providers, including positive effects of physical activity or nutritional recommendations, and the usual monitoring health care sessions, which were equal to the exercise group. They also were asked about their exercise once each trimester; to this end, a “decision algorithm” (by telephone) was used (Barakat et al., 2016).

Algorithm:

Question 1: Since the beginning of pregnancy, have you exercised in your leisure time, in a supervised program or on your own?
a. Answer: No.
b. Answer: Yes.
Question 2: (if the previous response was “b”): Given 7 days a week, how many days per week did you exercise?
a. Answer: Less than 3 days.
b. Answer: 3 days or more.
Question 3: (if the previous response was “b”): Considering the total duration of physical exercise continuously, how long did you exercise every day?
a. Answer: Less than 20 min each day.
b. Answer: 20 min or more each day.
Interpretation of the “Decision Algorithm”: Pregnant women in the CG who reached level b of these three questions, were excluded from the study.

### Outcomes

#### Primary Outcome

Data corresponding to SBP and DBP were obtained from protocol obstetric visits and delivery records following established hospital protocols. Therefore, data from the first, second and third trimesters as well as previous and immediate deliveries were collected. Blood pressure during pregnancy was recorded at week 10 ± 2 for the first trimester, at week 22 ± 1.5 for the second, and at week 36 ± 1.5 for the third trimester. Data collection of SBP and DBP of delivery was performed during the period of dilation, about an hour before childbirth and the postpartum measurement was taken immediately after delivery, in the “immediate puerperium.”

#### Secondary Outcomes

Gestational weight gain was calculated from the data of pregravid and last clinic visit before delivery weights. It was classified according to the 2009 Institute of Medicine (IOM) guidelines (Institute of Medicine (US) and National Research Council (US) Committee to Reexamine IOM Pregnancy Weight Guidelines, 2009). Excessive body weight gain was determined by IOM guidelines for pre-pregnancy BMI categories for each woman; >18 kg for underweight; >16 kg for normal; >11.5 kg for overweight; and >9 kg for obese women.

Data on maternal gestational age, birth weight, Apgar scores and pH of the umbilical cord blood were obtained from hospital perinatal records. Newborns were classified as having macrosomia when birth weight was >4,000 g and low birth weight was defined as <2,500 g.

### Statistical Analysis

Version 25.0 of IBM SPSS for Windows (IBM Corporation, Armonk, NY, United States) was used for all data analyses. Preliminary assessments were conducted using the Kolmogorov-Smirnov test to screen for violations of normality.

Pearson’s chi-square test was used to compare the obtained frequencies of maternal BMI, smoking, previous miscarriages, parity and employment occupation between the IG and CG.

Independent *t*-tests were used to assess the differences in age, gestational age, weight and height between the intervention and control groups.

In addition, this same test was used to examine SBP and DBP data in the first, second and third trimesters, previous delivery time and immediately after delivery between study groups. Besides, one Factor Repeated Measure ANOVA used to assess changes in SBP and BPD throughout pregnancy within the IG and CG and between both groups. Multiple comparisons were made using the Bonferroni test. The effect size was obtained using the partial index η^2^.

The data for continuous variables are presented as the means and standard deviations, and those of the nominal variables are presented as frequencies and percentages. The level of statistical significance was set at *p* < 0.05.

## Results

A total of 206 women over 18 years of age were randomized (ratio 1:1) in the study, and 84 were excluded: 41 did not meet the inclusion criteria, 8 declined to participate, and 35 declined for other reasons. Then, 122 women were randomized into IG (*n* = 61) and CG (*n* = 61). In the IG, 30 women were lost to follow-up: 3 had premature deliveries, 10 had low adherence, 8 had changes in hospital stay, and 9 had other reasons. The minimum adherence required has been attendance to 80% of the classes within the intervention group. In the CG, 20 women were lost to follow-up: 2 had persistent bleeding, 3 had premature delivery, 5 had changes in hospital stay, and 10 had other reasons. Finally, 31 women in the IG and 41 in the CG were analyzed (Figure 1).

Table 1 shows the general characteristics of the pregnant women in the study groups. No significant differences (*p* > 0.05) in maternal characteristics were found between the groups.

**TABLE 1:** Maternal characteristics.

Variable	Intervention group (*n* = 31)	Control group (*n* = 42)	*p-*values
Age (years)	32.29 ± 6.36	33.93 ± 4.59	0.205
Maternal height (m)	1.63 ± 0.05	1.63 ± 0.07	0.816
Maternal weight (kg)	60.37 ± 9.73	66.34 ± 14.12	0.059
BMI (*n*/%)	22.61 ± 3.22	23.06 ± 7.80	0.776
<18.5	2/7.1	4/9.8	0.190
18.5–24.9	22/78.6	22/53.7	
25–29.9	3/10.7	11/26.8	
>30	1/3.6	4/9.8	
**Parity^†^ (*n*/%)**			
None	25/80.6	27/65.9	0.333
One	5/16.1	10/24.4	
Two or more	1/3.2	4/9.8	
**Smoking during pregnancy**			
No	30/96.8	35/83.3	0.069
Yes	1/3.2	7/16.7	
**Occupation (*n*/%)**			
Active job	15/48.4	19/45.2	0.063
Sedentary job	13/41.9	11/26.2	
Homemaker	2/6.5	12/28.6	
**Previous miscarriage (*n*/%)**			
None	25/80.6	26/63.4	0.069
One	6/19.4	9/22.0	
Two or more	0/0	6/14.6	

**^†^Parity: children until current pregnancy.**

There were no significant differences in systolic and diastolic blood pressure at the first, second and third trimesters between the IG and CG (*p* > 0.05) (Table 2).

**TABLE 2:** Systolic blood pressure and diastolic blood pressure at first, second, and third trimester in IG and CG.

		Intervention group (*n* = 31)	Control group (*n* = 42)	*p-*values
First trimester	SBP	110.5512.13	110.7613.30	0.949
	DBP	71.007.41	72.958.07	0.317
Second trimester	SBP	108.8114.41	113.9615.46	0.220
	DBP	68.969.58	70.157.97	0.628
Third trimester	SBP	115.4511.86	116.4312.76	0.753
	DBP	74.556.53	73.7710.01	0.720

No significant intergroup differences were found (IG vs. CG) comparing the evolution of SBP [*F*_(3.78)_ = 0.639; *p* = 0.551; η^2^ = 0.018] and DBP [*F*_(3,10)_ = 0.684; *p* = 0.564; η^2^ = 0.020] during the whole process of pregnancy.

Performing an intragroup analysis, there were significant differences within the IG in the SBP value depending on the time of pregnancy [*F*_(2.36)_ = 3.086; *p* = 0.046; η^2^ = 0.128]. Multiple comparisons show that SBP in the third trimester was significantly higher compared to the second trimester (*p* = 0.022). However, no differences were found between the first trimester in relation to the second and third. On the other hand, within the CG, no significant differences were found in the evolution of SBP [*F*_(1.30)_ = 1.65; *p* = 0.210; η^2^ = 0.089].

In relation to DBP, significant differences were found depending on the time of pregnancy within the IG [*F*_(2.42)_ = 4.34; *p* = 0.019; η^2^ = 0.171] as of the CG [*F*_(2.34)_ = 4.37; *p* = 0.020; η^2^ = 0.205].

Within the IG, DBP showed a significant increase in the third trimester of pregnancy compared to the second (*p* = 0.036). No significant differences were found between the first compared to the second and third trimesters of pregnancy (*p* > 0.05).

Finally, within the CG, multiple comparisons revealed that DBP presented a significant decrease in the second trimester of pregnancy compared to the first (*p* = 0.007). At last, no significant differences were observed between the third trimester compared to the first and second.

According to the analysis, significant differences in SBP were found immediately before delivery (*t*_56_ = 2.034; IG = 119.83 ± 10.16 vs. CG = 125.6 ± 10.91; *p* = 0.047) and immediately after delivery (*t*_60_ = 2.046; IG = 115.00 ± 11.18 vs. CG = 122.24 ± 15.71; *p* = 0.045). There were no significant differences in DBP between the IG and CG either immediately before delivery (IG = 72.82 ± 8.20 vs. CG = 73.94 ± 9.88; *p* = 0.63) or immediately after delivery (IG = 65.71 ± 9.14 vs. CG = 68.47 ± 13.62; *p* = 0.329).

## Discussion

We examined the effects of virtual exercise programs during pregnancy on the control of blood pressure in healthy pregnant women. This novel approach used an integration of light resistance, toning, aerobic dance, stretching, and pelvic floor exercises in the virtual training program, which were easily incorporated into a structured exercise regime. Although not analyzed in this study, this new program includes special care for the emotional aspect of pregnancy; this aspect is generally not available to the pregnant population in exercise programs. It seems that this program was equally liked by all BMI categories, as indicated by the high adherence rate.

Our intervention exercise during pregnancy does not affect the values obtained from the protocol for obstetric controls of blood pressure in the first, second and third trimesters; however, in the data prior to and immediately after delivery, a decrease in SBP of the IG was found. Despite finding statistically significant differences in SBP and BPD within the IG and CG, these differences are within the normal levels of obstetric control and the evolution of this parameter during pregnancy.

Both time points (before and after delivery) are especially relevant due to the hemodynamic stress to which the pregnant woman is subjected to. These results may suggest a “decompression” effect of the maternal circulatory system during stressful events. From a hemodynamic point of view, the decrease in SBP in the IG group at delivery, but not in the 1st, 2nd, or 3rd trimesters, could be due to effects based on the adaptation to the acute requirements of the maternal circulatory system that exercise training during pregnancy may perpetuate (Haakstad et al., 2016).

No cause was identified for cardiovascular disorders, such as pregnancy-induced hypertension. However, it seems to be developed early in gestation, appearing symptoms in the mid- to late part of the pregnancy (Roberts and Lain, 2002). Important advances in this field have been published recently, including the identification of long-term maternal and fetal risks conferred by pre-eclampsia (Phipps et al., 2019). This could be enhanced by the complex situation caused by COVID-19 (confinement and movement restrictions, social isolation, etc.).

The benefits of an exercise program throughout pregnancy on SBP before and after delivery in healthy pregnant women (also inducing a healthy lifestyle) could demonstrate the need to promote new strategies (an exercise program) and might be key issues to prevent chronic disease risk.

From a scientific point of view, epidemiological studies suggest that women who are physically active are less likely to develop gestational hypertension (Sorensen et al., 2003; Saftlas et al., 2004; Rudra et al., 2008; Martin and Huber, 2010; Barakat et al., 2016), although new data regarding the relationship between exercise during pregnancy and maternal blood pressure in the current COVID-19 pandemic situation are urgently needed.

From our point of view, this is the first study linking an early exercise intervention with high adherence to reduce the values of maternal SBP before and after delivery.

The scientific literature confirms that maternal exercise during pregnancy has important benefits; it has been associated with lowering blood pressure (Yeo, 2010), and the effects of exercise include an increase in aerobic and cardiovascular conditioning (Ruchat et al., 2012). Exercise may protect against preeclampsia by reducing maternal byproducts of oxidative stress, preventing endothelial dysfunction, and stimulating vascularity and placental growth (Falcao et al., 2010).

In addition, exercise has been linked to beneficial fetal and pregnancy outcomes (Wang et al., 2015; Pelaez et al., 2019; Vargas-Terrones et al., 2020), including mental and emotional aspects, which affect maternal blood pressure.

Our large RCT confirms the beneficial effects of exercise during pregnancy on the control of maternal blood pressure and demonstrates the importance of the use of the lifestyle factors of pregnant women for the prevention of risk factors and cardiovascular diseases during the pandemic state. In summary, a healthy lifestyle intervention with high adherence could be a relevant preventive element in the health of the pregnant population. It is important to note that the results could be affected by the nature of the follow-up used and the exclusions in both study groups.

## Conclusion

A virtual exercise program throughout pregnancy during COVID-19 confinement can help to control systolic blood pressure before and immediately after delivery in healthy pregnant women.

### Strengths and Weaknesses

The main strengths of our study are the design and development of a large virtual RCT with high adherence (≥80% attendance) in our exercise group, while also examining the activity of the pregnant women in the CG. We believe that this is especially relevant in the current pandemic situation.

Assessment of the exercise activity of CG (excluding highly active women) and evaluation of adherence (attendance) to a virtual exercise program of IG must be considered when examining the effects of RCTs to provide relevant information from the primary outcome to clinical practice.

Although we did not specifically evaluate women with elevated hypertension or preeclampsia, the fact that women who exercised had lower SBP values (a precursor to the development of preeclampsia) also suggests that maternal exercise may prevent cardiovascular complications.

A possible limitation of our study has been the exclusion from the final analysis of women who were non-adherent (IG) and women who were physically active (CG). Besides, other limitation of our study is the lack of nutrition or energy intake assessment; however, all participants received standard care and information about a healthy lifestyle during pregnancy in their formal obstetric consults.

In addition, the supervision of a virtual program is not identical to a face-to-face session; however, this is due to the current pandemic situation and thus using online technologies is the only way in which health authorities will allow this type of intervention with a pregnant population (program exercise). Finally, another limitation could have been the of the lack of data collection for family history of hypertension, and this could be an interesting variable to analyze in future studies.

Therefore, due to the complex healthcare environment caused by COVID-19, future studies should examine the preventive effects of interventions in alterations that the current pandemic situation will cause.

## Data Availability Statement

The raw data supporting the conclusions of this article will be made available by the authors, without undue reservation.

## Ethics Statement

The studies involving human participants were reviewed and approved by the Ethical Commission of Research of Universidad Politécnica de Madrid. The patients/participants provided their written informed consent to participate in this study.

## Author Contributions

CS-J and MS-P were responsible for the design and development of the virtual supervised program and contributed to the data analysis and the preparation of the manuscript. ÁD-B contributed to the recruitment and randomization of pregnant women, clinical data collection, registration, and analysis. JC and RB contributed to the experimental design, data analysis, and preparation of the manuscript. IR oversaw the experimental design, data analysis, and manuscript writing. All authors contributed to the article and approved the submitted version.

## Conflict of Interest

The authors declare that the research was conducted in the absence of any commercial or financial relationships that could be construed as a potential conflict of interest.

## References

[B1] Acog Committee on Practice Bulletins–Obstetrics (2002). ACOG practice bulletin. diagnosis and management of preeclampsia and eclampsia. Number 33, January 2002. *Obstet. Gynecol.* 99 159–167. 10.1016/s0029-7844(01)01747-1 16175681

[B2] Alomari M. A. Khabour O. F. Alzoubi K. H. (2020). Changes in physical activity and sedentary behavior amid confinement: the BKSQ-COVID-19 project. *Risk Manag. Healthc. Policy* 13 1757–1764. 10.2147/RMHP.S268320 33061709 PMC7526007

[B3] Ammar A. Brach M. Trabelsi K. Chtourou H. Boukhris O. Masmoudi L. (2020a). Effects of COVID-19 home confinement on eating behaviour and physical activity: results of the ECLB-COVID19 international online survey. *Nutrients.* 12:1583. 10.3390/nu12061583 32481594 PMC7352706

[B4] Ammar A. Trabelsi K. Brach M. Chtourou H. Boukhris O. Masmoudi L. (2020b). Effects of home confinement on mental health and lifestyle behaviours during the COVID-19 outbreak: insight from the ECLB-COVID19 multicenter study. *Biol. Sport* 38 9–21. 10.5114/biolsport.2020.96857 PMC7996377 33795912

[B5] Amro F. Sibai B. (2020). Management of hypertension in pregnancy. *Semin. Fetal Neonatal Med.* 25:101147. 10.1016/j.siny.2020.101147 33121915

[B6] Artal R. Wiswell R. Drinkwater B. (1991). *Exercise in Pregnancy*, 2nd Edn. Baltimore, MD: Williams and Wilkins.

[B7] Ayaz R. Hocaoğlu M. Günay T. Yardımcı O. D. Turgut A. Karateke A. (2020). Anxiety and depression symptoms in the same pregnant women before and during the COVID-19 pandemic. *J. Perinat. Med.* 48 965–970. 10.1515/jpm-2020-0380 32887191

[B8] Bamfo J. E. A. K. Kametas N. A. Nicolaides K. H. Chambers J. B. (2007). Maternal left ventricular diastolic and systolic long-axis function during normal pregnancy. *Eur. J. Echocardiogr.* 8 360–368. 10.1016/j.euje.2006.12.004 17321800

[B9] Barakat R. (2020). An exercise program throughout pregnancy: Barakat model. *Birth Defects Res.* 113 218–226. 10.1002/bdr2.1747 32613735

[B10] Barakat R. Díaz-Blanco A. Franco E. Rollán-Malmierca A. Brik M. Vargas M. (2019). Guías clínicas para el ejercicio físico durante el embarazo. *Prog. Obstet. Ginecol.* 62 464–471. 10.20960/j.pog.00231 27759997

[B11] Barakat R. Pelaez M. Cordero Y. Perales M. Lopez C. Coteron J. (2016). Exercise during pregnancy protects against hypertension and macrosomia: randomized clinical trial. *Am. J. Obstet. Gynecol.* 214:649.e1–.e8. 10.1016/j.ajog.2015.11.039 26704894

[B12] Barakat R. Perales M. Garatachea N. Ruiz J. R. Lucia A. (2015). Exercise during pregnancy. a narrative review asking: what do we know? *Br. J. Sports Med.* 49 1377–1381. 10.1136/bjsports-2015-094756 26135742

[B13] Bellamy L. Casas J. P. Hingorani A. D. Williams D. J. (2007). Pre-eclampsia and risk of cardiovascular disease and cancer in later life: systematic review and meta-analysis. *BMJ* 335:974. 10.1136/bmj.39335.385301.BE 17975258 PMC2072042

[B14] Carbillon L. Uzan M. Uzan S. (2000). Pregnancy, vascular tone, and maternal hemodynamics: a crucial adaptation. *Obstet. Gynecol. Surv.* 55 574–581. 10.1097/00006254-200009000-00023 10975484

[B15] Chivers B. R. Garad R. M. Boyle J. A. Skouteris H. Teede H. J. Harrison C. L. (2020). Perinatal distress during COVID-19: thematic analysis of an online parenting forum. *J. Med. Internet. Res.* 22:e22002. 10.2196/22002 32857707 PMC7481017

[B16] Clapp J. F. III Capeless E. (1997). Cardiovascular function before, during, and after the first and subsequent pregnancies. *Am. J. Cardiol.* 80 1469–1473. 10.1016/s0002-9149(97)00738-8 9399724

[B17] Davenport M. H. Ruchat S. M. Poitras V. J. Jaramillo Garcia A. Gray C. E. Barrowman N. (2018). Prenatal exercise for the prevention of gestational diabetes mellitus and hypertensive disorders of pregnancy: a systematic review and meta-analysis. *Br. J. Sports. Med.* 52 1367–1375. 10.1136/bjsports-2018-099355 30337463

[B18] Durankuş F. Aksu E. (2020). Effects of the COVID-19 pandemic on anxiety and depressive symptoms in pregnant women: a preliminary study. *J. Matern. Fetal. Neonatal. Med.* 18 1–7. 10.1080/14767058.2020.1763946 32419558

[B19] Falcao S. Bisotto S. Michel C. Lacasse A. A. Vaillancourt C. Gutkowska J. (2010). Exercise training can attenuate preeclampsia-like features in an animal model. *J. Hypertens.* 28 2446–2453. 10.1097/HJH.0b013e32833e97d0 20811291

[B20] Geva T. Mauer M. B. Striker L. Kirshon B. Pivarnik J. M. (1997). Effects of physiologic load of pregnancy on left ventricular contractility and remodeling. *Am. Heart J.* 133 53–59. 10.1016/s0002-8703(97)70247-3 9006290

[B21] Gilson G. J. Samaan S. Crawford M. H. Qualls C. R. Curet L. B. (1997). Changes in hemodynamics, ventricular remodeling, and ventricular contractility during normal pregnancy: a longitudinal study. *Obstet. Gynecol.* 89 957–962. 10.1016/s0029-7844(97)85765-1 9170474

[B22] Haakstad L. A. Edvardsen E. Bø K. (2016). Effect of regular exercise on blood pressure in normotensive pregnant women. A randomized controlled trial. *Hypertens. Pregnancy* 35 170–180. 10.3109/10641955.2015.1122036 26909888

[B23] Institute of Medicine (US) and National Research Council (US) Committee to Reexamine IOM Pregnancy Weight Guidelines. (2009). “Weight gain during pregnancy,” in *Reexamining the Guidelines*, eds Rasmussen K. M. Yaktine A. L. (Washington, DC: National Academies Press). 20669500

[B24] Juan J. Gil M. M. Rong Z. Zhang Y. Yang H. Poon L. C. (2020). Effect of coronavirus disease 2019 (COVID-19) on maternal, perinatal and neonatal outcome: systematic review. *Ultrasound Obstet. Gynecol.* 56 15–27. 10.1002/uog.22088 32430957 PMC7276742

[B25] Justman N. Shahak G. Gutzeit O. Ben Zvi D. Ginsberg Y. Solt I. (2020). Lockdown with a price: the impact of the COVID-19 pandemic on prenatal care and perinatal outcomes in a tertiary care center. *Isr. Med. Assoc. J.* 22 533–537. 33236549

[B26] Magee L. A. Khalil A. von Dadelszen P. (2020). Pregnancy hypertension diagnosis and care in COVID-19 era and beyond. *Ultrasound Obstet. Gynecol.* 56 7–10. 10.1002/uog.22115 32506723 PMC7300934

[B27] Martin C. L. Huber L. (2010). Physical activity and hypertensive complications during pregnancy: findings from 2004 to 2006 North Carolina pregnancy risk assessment monitoring system. *Birth* 37 202–210. 10.1111/j.1523-536X.2010.00407.x 20887536

[B28] Mottola M. F. Davenport M. H. Ruchat S. M. Davies G. A. Poitras V. J. Gray C. E. (2018). 2019 Canadian guideline for physical activity throughout pregnancy. *Br. J. Sports Med.* 52 1339–1346. 10.1136/bjsports-2018-100056 30337460

[B29] Moyer C. A. Compton S. D. Kaselitz E. Muzik M. (2020). Pregnancy-related anxiety during COVID-19: a nationwide survey of 2740 pregnant women. *Arch. Womens Ment. Health* 23 757–765. 10.1007/s00737-020-01073-5 32989598 PMC7522009

[B30] Obstetrics and Gynecology (2013). Hypertension in pregnancy. Report of the American College of Obstetricians and Gynecologists’ Task Force on Hypertension in Pregnancy. *Obstet. Gynecol.* 122 1122–1131. 10.1097/01.AOG.0000437382.03963.88 24150027

[B31] Obstetrics and Gynecology (2019). ACOG Practice Bulletin No. 202: Gestational Hypertension and Preeclampsia. *Obstet. Gynecol.* 133:1. 10.1097/AOG.0000000000003018 30575675

[B32] Obstetrics and Gynecology (2020). Physical Activity and Exercise During Pregnancy and the Postpartum Period: ACOG Committee Opinion, Number 804. *Obstet. Gynecol.* 135 e178–e188. 10.1097/AOG.0000000000003772 32217980

[B33] Pelaez M. Gonzalez-Cerron S. Montejo R. Barakat R. (2019). Protective effect of exercise in pregnant women including those who exceed weight gain recommendations: a randomized controlled trial. *Mayo Clin. Proc.* 94 1951–1959. 10.1016/j.mayocp.2019.01.050 31585579

[B34] Perales M. Santos-Lozano A. Sanchis-Gomar F. Luaces M. Pareja-Galeano H. Garatachea N. (2016). Impact of gestational risk factors on maternal cardiovascular system. *Ann. Transl. Med.* 4:253. 10.21037/atm.2016.06.18 27500154 PMC4958727

[B35] Phipps E. A. Thadhani R. Benzing T. Karumanchi S. A. (2019). Author correction: pre-eclampsia: pathogenesis, novel diagnostics and therapies. *Nat. Rev. Nephrol.* 15:386. 10.1038/s41581-019-0156-1 31068691

[B36] Rand B. G. Johnson T. M. Ehrlich S. F. Wideman L. Pivarnik J. M. Richardson M. R. (2020). Diabetes risk status and physical activity in pregnancy: U.S. BRFSS 2011, 2013, 2015, 2017. *BMC Pregnancy Childbirth* 20:743. 10.1186/s12884-020-03434-5 33256646 PMC7708155

[B37] Roberts J. M. Lain K. Y. (2002). Recent Insights into the pathogenesis of pre-eclampsia. *Placenta* 23 359–372. 10.1053/plac.2002.0819 12061851

[B38] Ruchat S. M. Davenport M. H. Giroux I. Hillier M. Batada A. Sopper M. M. (2012). Walking program of low or vigorous intensity during pregnancy confers an aerobic benefit. *Int. J. Sports Med.* 33 661–666. 10.1055/s-0032-1304635 22510805

[B39] Rudra C. B. Sorensen T. K. Luthy D. A. Williams M. A. (2008). A prospective analysis of recreational physical activity and preeclampsia risk. *Med. Sci. Sports Exerc.* 40 1581–1588. 10.1249/MSS.0b013e31817cab1 18685534

[B40] Saftlas A. F. Logsden-Sackett N. Wang W. Woolson R. Bracken M. B. (2004). Work, leisure-time physical activity, and risk of preeclampsia and gestational hypertension. *Am. J. Epidemiol.* 160 758–765. 10.1093/aje/kwh277 15466498

[B41] Sorensen T. K. Williams M. A. Lee I. M. Dashow E. E. Thompson M. L. Luthy D. A. (2003). Recreational physical activity during pregnancy and risk of preeclampsia. *Hypertension* 41 1273–1280. 10.1161/01.HYP.0000072270.82815.91 12719446

[B42] Vargas-Terrones M. Nagpal T. S. Perales M. Prapavessis H. Mottola M. F. Barakat R. (2020). Physical activity and prenatal depression: going beyond statistical significance by assessing the impact of reliable and clinical significant change. *Psychol. Med.* 27 1–6. 10.1017/S0033291719003714 32102723

[B43] Wang C. Zhu W. Wei Y. Feng H. Su R. Yang H. (2015). Exercise intervention during pregnancy can be used to manage weight gain and improve pregnancy outcomes in women with gestational diabetes mellitus. *BMC Pregnancy Childbirth* 15:255. 10.1186/s12884-015-0682-1 26459271 PMC4603976

[B44] Yeo S. (2010). Prenatal stretching exercise and autonomic responses: preliminary data and a model for reducing preeclampsia. *J. Nurs. Scholarsh.* 42 113–121. 10.1111/j.1547-5069.2010.01344.x 20618595 PMC2904621

[B45] Yong H. Y. Shariff Z. M. Mohd Yusof B. N. Rejali Z. Bindels J. Tee Y. Y. S. (2020). High physical activity and high sedentary behavior increased the risk of gestational diabetes mellitus among women with excessive gestational weight gain: a prospective study. *BMC Pregnancy Childbirth* 20:597. 10.1186/s12884-020-03299-8 33028258 PMC7541260

[B46] Zaigham M. Andersson O. (2020). Maternal and perinatal outcomes with COVID-19: a systematic review of 108 pregnancies. *Acta Obstet. Gynecol. Scand.* 99 823–829. 10.1111/aogs.13867 32259279 PMC7262097

[B47] Zentner D. Wheeler M. Grigg L. (2012). Does pregnancy contribute to systemic right ventricular dysfunction in adults with an atrial switch operation? *Heart Lung Circ.* 21 433–438. 10.1016/j.hlc.2012.04.009 22578588

